# Observer blind randomised controlled trial of a tailored home exercise programme versus usual care in people with stable inflammatory immune mediated neuropathy

**DOI:** 10.1186/s12883-015-0398-x

**Published:** 2015-08-21

**Authors:** Claire M. White, Robert D. Hadden, Sarah F. Robert-Lewis, Paul R. McCrone, Jane L. Petty

**Affiliations:** Division of Health and Social Care, King’s College London, London, SE1 1UL UK; Department of Neurology, King’s College Hospital, London, UK; Centre for the Economics of Mental and Physical Health, Institute of Psychiatry, King’s College London, London, UK

**Keywords:** Inflammatory neuropathy, Activity limitation, Home exercise, Observer blind randomised controlled trial

## Abstract

**Background:**

Inflammatory neuropathies such as Guillain-Barré syndrome, chronic inflammatory demyelinating polyradiculoneuropathy and paraproteinaemic demyelinating neuropathy are a heterogenous group of peripheral nerve disorders that affect around one to two people per 100,000. Whilst treatments such as intravenous immunoglobulin, plasma exchange and corticosteroids have generally positive results, long-term residual symptoms and associated activity limitations are common.

There is currently no standardised care for patients with ongoing activity limitation and participation restriction as a result of inflammatory neuropathy IN but data from observational studies and a randomised controlled trial suggest that exercise either alone or as part of a multidisciplinary rehabilitation programme may be beneficial in improving activity limitation. Tailoring the intervention for participants following physiotherapy assessment and incorporating patient preference for type and location of exercise may be important.

**Methods/Design:**

The current study is a pragmatic, prospective, parallel observer-blind, randomised controlled trial to evaluate the efficacy and cost-effectiveness of a twelve week tailored home exercise programme versus advice and usual care. Seventy adults with stable immune mediated inflammatory neuropathy IN will be recruited to the study from two main sources: patients attending selected specialist peripheral nerve clinics in the South East and West Midlands of England and people with who access the GAIN charity website or newsletter. Participants will be randomised to receive either advice about exercise and usual care or a 12 week tailored home exercise programme.

The primary outcome of activity limitation and secondary outcomes of fatigue, quality of life, self-efficacy, illness beliefs, mood and physical activity will be assessed via self-report questionnaire at baseline, 12 weeks and 12 months post intervention. Cost effectiveness and cost utility will be assessed via interview at baseline and 12 months post intervention.

Intention to treat analysis will be our primary model for efficacy analysis. Semi-structured interviews will be conducted with a selected sample of participants in order to explore the acceptability of the intervention and factors affecting adherence to the exercise programme.

**Discussion:**

This is the first randomised controlled trial to compare the efficacy and cost-effectiveness of tailored home exercise with advice about exercise and usual care for adults with inflammatory neuropathy.

**Trial registration:**

Current Controlled Trials ISRCTN13311697

## Background

Inflammatory immune mediated neuropathies (IN) are a heterogenous group of peripheral nerve disorders that affect around one to two people per 100,000 [1]. The conditions share an immune-mediated pathophysiological process but the clinical features may vary between conditions. Guillain-Barré syndrome (GBS) is an acute inflammatory polyneuropathy, characterised by rapidly progressive weakness, paralysis and sensory impairment reaching nadir within four weeks of onset [2] such that the majority of patients are unable to walk independently when maximum weakness is reached [3]. Prompt immunomodulatory treatment facilitates earlier recovery that is generally good; with the majority of patients regaining mobility within six to twelve months [4, 5] although residual problems are common and improvements may continue beyond this period [6]. Chronic inflammatory demyelinating polyradiculoneuropathy (CIDP) shares many characteristics with GBS but presents a progressive or relapsing clinical course [7]. There are a number of first-line, combination and supportive treatments available for CIDP, [8] and whilst many patients experience long-term improvement, prognosis is variable and a significant proportion experience ongoing problems [4]. A related condition, paraproteinaemic demyelinating neuropathy (PDN), is characterised by an IgM monoclonal gammopathy with, in about 50 % of cases, serum antibodies to myelin associated glycoprotein. PDN typically presents at an older age than GBS and CIDP, with most cases presenting in the sixth decade or later and symptoms appear similar to CIDP [4]. Despite differences in the initial presentation and time course of GBS, CIDP and PDN, the symptoms experienced by patients and the impact of them on their daily lives are similar.

**Fig. 1:**
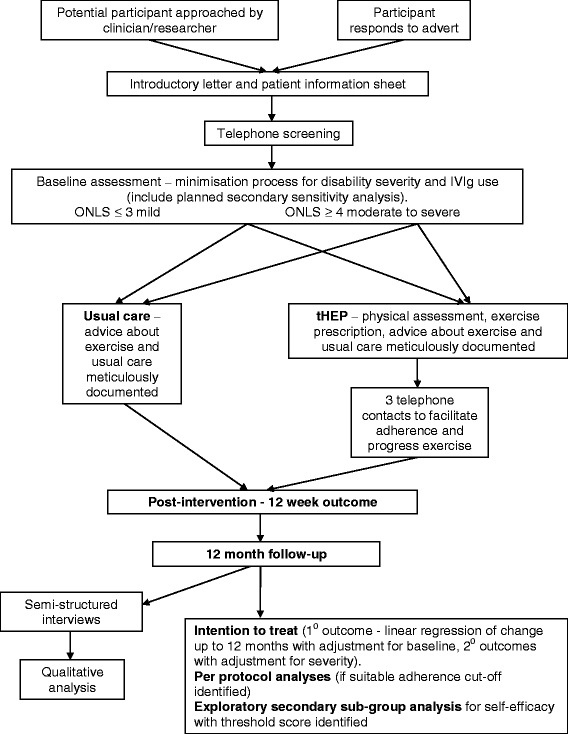
Study design flowchart. Flowchart depicting the process of recruitment, randomisation, data collection and data analysis throughout the trial

At least a third of people with IN experience long term activity limitation. Residual symptoms may still be present many years after recovery from GBS [9–12] and CIDP [13]. One ten year follow-up study [14] showed that 14 % of participants with GBS still had moderate to severe disability and a further 50 % had more minor symptoms, suggesting that residual disability may be life-long. Fatigue is also a very common consequence of IN with between 40 – 80 % of people reporting it [15, 16]. It is often associated with reduced quality of life [15] and greater activity limitation [14].

Persistent disability or fatigue experienced by people with IN adversely affects their working and daily lives, including family and social activities [9, 12, 13, 17–19]. Many people with IN need long-term informal care, often supplemented by support from health and social services [20]. People with IN are high users of services [21] and the personal suffering, as well as health and social care expenditure, increases as people live longer. Therefore, the potential for cost effective self-management programmes aimed at improving disability, mood, societal participation and quality of life should be evaluated.

There is currently no standardised care for patients with ongoing activity limitation and participation restriction as a result of IN. A systematic review of the efficacy of exercise on disability in IN concluded that the current evidence is of poor quality, although progressive resisted strengthening exercise may moderately improve muscle strength [22]. However, observational studies show that supervised aerobic-cycling [23] or unsupervised physiotherapist prescribed community based aerobic and strengthening exercise [24] are associated with an improvement in function, fatigue and quality of life in people with IN. Further, one randomised controlled trial showed that high intensity multi-disciplinary rehabilitation that incorporated physiotherapy for strengthening, endurance and gait training was superior to low-intensity rehabilitation in improving disability in people with GBS several years after recovery [25]. Therefore exercise may be beneficial in improving activity limitation after IN and tailoring the intervention for participants following physiotherapy assessment and incorporating patient preference for type and location of aerobic and strengthening exercise may be important [24].

A tailored approach to exercise prescription based on our pilot study [24] has been developed for the current study in light of social cognition theory [26]. The theory suggests that behaviour is determined by the interaction of current and previous behaviour, social, environmental and cognitive factors such as social support, observational learning or modelling, outcome expectations, self-efficacy, goal setting and self-regulation.

Thus, the current pragmatic randomised controlled trial (RCT) includes a process evaluation that is underpinned by the transtheoretical model (TTM) for behaviour change [27]. The TTM has been shown to be useful in understanding and predicting behaviour change across a range of health behaviours including physical activity [28, 29]. The model proposes a temporal dimension of stages of change (SoC) to describe the sequential change in health related behaviours. The five stages of change are: pre-contemplation, contemplation, preparation, action and maintenance [27, 30]. The model assumes that the likelihood for behavioural change will be dependent on the stage of change at which individuals are currently at and that progress through the stages may be complex. Factors such as self-efficacy and decisional balance (the relative pros and cons of behaviour change) have been consistently shown to increase in a linear fashion alongside this progression through stages [31], suggesting various potential factors to be targeted for intervention.

In order to evaluate the potential role of home exercise for the management of IN, the current RCT will therefore assess the effectiveness, cost effectiveness and cost utility of a tHEP versus advice about exercise and usual care (UC) in a sample of adults with stable IN in the UK.

### Objectives and hypotheses

#### Principal research question

Is a tailored home exercise programme more effective in reducing disability (activity limitation) than advice about exercise and usual care in people with stable inflammatory immune-mediated neuropathy?

Primary hypothesis - a tailored home exercise programme will be more beneficial than advice about exercise and usual care in reducing activity limitation for people with IN.

#### Secondary research questions

What is the cost-effectiveness and cost utility of a tHEP compared with advice about exercise and usual care for people with IN?

Are the health beliefs of people with IN associated with their response to a tHEP versus advice about exercise and usual care?

What are participants’ experiences of a tHEP and which factors influence uptake of and adherence to the programme?

## Methods

### Design

The study is a pragmatic, prospective, parallel observer blind, RCT to evaluate the efficacy and cost-effectiveness of a twelve week tHEP versus advice and usual care. Assessments will be taken at baseline, immediately following the intervention (twelve weeks) and at twelve months. See Fig. 1. The single outcome assessor will remain blinded until the end of the twelve month follow-up period but due to the nature of the intervention, the participants and research physiotherapist will not be blind to allocation.

### Participants

Seventy adults with stable immune mediated IN will be recruited to the study from two main sources: patients attending selected specialist peripheral nerve clinics in the South East and West Midlands of England and people with IN who access the Guillain-Barré syndrome and Associated Inflammatory Neuropathy (GAIN) charity website or newsletter. Potential participants from clinics will be approached by either their doctor or a member of the research team (with the prior consent of the patient’s doctor) and provided with information about the study. Alternatively, they will be sent an invitation letter and information sheet through the post. In both cases, participants will be offered the opportunity to discuss the project and ask questions. Signed consent forms will be returned directly to the research team if they decide to take part, either in person at the clinic, or via the post once the participant has returned home. Potential participants who access GAIN publications will be approached by advertisements on their website and newsletters. Potential participants will contact the research team directly to receive information sheets and to discuss the study prior to consenting to take part.

All potential participants will be screened for compliance with the study inclusion criteria (below) and their diagnosis, IVIg therapy and fitness to participate will be confirmed with their GP or neurologist, with their consent.

### Inclusion/exclusion criteria

Participants will be included if they fulfil the following criteria:

1. They are adults with stable motor neuropathy, with or without sensory neuropathy, as a result of GBS, CIDP or PDN diagnosed using established criteria [32, 33] (and where PDN is defined as the combination of demyelinating neuropathy, serum antibodies to myelin associated glycoprotein, and an IgM monoclonal gammopathy with no evidence of haematological malignancy)
2. They are able to walk 10 m, with or without walking aids
3. They are at least one year since onset if they have GBS
4. They have had no change in self-reported disability, immunotherapy or medication for neuropathic pain in the previous six months (excepting medication dose of azathioprine, which must not have changed for twelve months). Patients receiving regular intravenous immunoglobulin (IVIg) or plasma exchange will be assessed at the same time points after treatment to avoid fluctuations due to time since last treatment. Minor alterations to IVIg therapy in the lead-up to the project will be allowed. A < =10 % change in the average weekly dose will be allowed in the 3 months prior to entry to the trial, and a change of < =20 % in the previous 6 months. If patients have received greater changes, they will still be able to take part but must wait until they meet these criteria
5. Participants should not have received physiotherapy treatment in the six months prior to entering the study. Once in the trial, patients may receive additional physiotherapy for other unrelated musculoskeletal problems if necessary but where possible this should be postponed, at least until after the twelve week intervention phase
6. They are able to understand spoken and/or written English and are able to communicate responses to questionnaires

Participants will be excluded from the study if they fulfil any of the following criteria:

1. They score zero on the overall neuropathy limitations scale (ONLS) [34], or 1 on the upper limb scale alone (as this would reflect sensory symptoms not affecting function)
2. They have any other unstable medical conditions that a) affect activity limitation b) prevents them from exercising or c) would make it unsafe to exercise
3. They are pregnant
4. They are adults who are unable to consent for themselves

### Sample size

The sample size is based on an 80 % power calculation to detect a difference between mean change in the overall disability sum score (ODSS) [35] of 1 point using a 2 sided test at the 5 % significance level based on a SD of 1.27 from pilot study data [24]. Fifty-four people (27 per group) will be needed; therefore 70 people will be recruited to allow for a 25 % attrition rate at twelve months. The ODSS is a pre-cursor of the ONLS and was used for sample size calculation as this was the recommended outcome measure of disability in IN at the time of the pilot study.

### Randomisation and allocation concealment

Participants will be allocated into either the treatment or control group using randomisation with computerised block minimisation to stratify for severity based on baseline ONLS score (mild ≤ 3, moderate/severe ≥ 4) and recruitment site to ensure even distribution between groups. Participants will also be stratified by IVIg treatment (yes/no) to minimise the likelihood that small numbers of participants receiving this expensive therapy may skew the cost-effectiveness analysis if not controlled for across the groups. Randomisation will be conducted by the King’s College London Clinical Trials Unit to ensure both the validity and balance of the randomisation and support the successful blinding of the outcome assessor for the intervention duration. Once allocation has taken place, the outcome observer will receive a blinded email confirming the participant number, and the research physiotherapist will receive an un-blinded email confirming participant number and allocation. In order to maintain observer blindness throughout the study period, participants will be requested not to discuss the intervention with the outcome assessor.

### Interventions

#### Control group

Participants allocated to the control group will receive by post a letter advising them that they have been allocated to the information and advice group and including the UK Physical Activity Guidelines factsheet outlining the benefits of regular exercise and the recommended level of exercise for adults or older adults [36] and a copy of the Physical Activity Readiness Questionnaire (PAR-Q) [37]. This group will not receive any contact with the study physiotherapist. They will receive any other usual medical care which may include pharmacological, physical or other therapy interventions deemed necessary by their doctor during the study period. Participants will be requested to try and avoid having additional physiotherapy for their IN during the study but treatment not directly related to their IN will not be restricted.

All referrals to other therapies and the specific interventions applied will be meticulously documented in both groups using the Client Service Receipt Inventory (CSRI) [38]. Data will include service utilisation in the preceding month (home-help, personal and informal care etc.) and less regularly used services in the preceding twelve months (General Practitioner (GP) visits, hospital admission etc.). Informal care and financial costs borne by carers will also be estimated and recorded.

#### Intervention group

In addition to usual care (as characterised above) the intervention group will be prescribed tHEP by the study physiotherapist. Participants will undergo a clinical interview and objective evaluation of their symptoms, activity limitations and any underlying impairments likely to affect their ability to exercise. In addition, in order to foster uptake and adherence to exercise behaviour, the therapist will ask participants to identify their preferences for exercise type and location and any factors that they feel may affect their willingness or ability to carry out a tHEP and how these may be overcome. This information will be used to facilitate the development of individualised exercise programmes tailored to take account of participants SoC and personal beliefs and that include physical activity and exercise goals identified by participants. Based on this assessment, the physiotherapist will prescribe a tailored progressive programme of aerobic and three strengthening exercises. Demonstration and practice of each exercise will ensure participants are carrying them out effectively, safely and with the confidence to continue exercising alone. Participants will be asked to continue the programme by themselves at home for twelve weeks. The therapist will advise participants on how and when to progress their exercises and this will be reinforced in follow-up telephone calls.

Aerobic exercise prescription will be informed by previous neurological exercise interventions [23, 24], evidence based exercise guidelines [39, 40] and participant preference. The dosage of exercise prescribed will be based on the recommendations of the UK Activity Guidelines [36]. Thus participants will be encouraged to gradually build up to at least the minimum recommended guideline amount of 150 min of moderate exercise a week. Moderate intensity exercise such as brisk walking or cycling should make adults feel as if they are breathing harder and their heart is beating faster but that they are still able to carry on a conversation. Participants will be taught how to use the Category Ratio 10 scale (CR-10) for rating of perceived exertion (RPE) [41] to evaluate exercise intensity where moderate intensity is graded at CR-10 grade 4.

Participants already meeting the guidelines at a moderate level, or those participants who build up to this level during the intervention period will be encouraged to set goals to either increase the exercise duration or to increase the intensity of exercise to a vigorous level [42]. Vigorous intensity exercise such as running or sporting activity, will make adults feel they are breathing much harder, their hearts are beating rapidly and they will find it more difficult to carry on a conversation. This intensity of exercise is graded 7 on the CR-10 of RPE [41].

Each participant will also be prescribed three strengthening exercises that target weak muscles, chosen from a standardised menu of strengthening exercises compiled by the study physiotherapist and informed by the literature and evidence based exercise guidelines [24, 40, 43, 44]. Each exercise, and any modifications or progressions, is facilitated by an exercise sheet supplied to the participant, with written and pictorial instructions based on those available on the peer reviewed website www.physiotherapyexercises.com [45]. In order to standardise exercise intensity participants will be advised to perform strengthening exercises at a RPE of 7–10 on the CR-10 [41, 46] with the goal of achieving 3 sets of 8–10 repetitions of each exercise prior to progression. Altering starting position may be used to eliminate or increase the influence of gravity on muscle activity and adding external resistance such as weighted bands or elastic resistance bands, may be used to increase the intensity of exercise as required. Examples of exercises include: heel raises in standing, squats and knee extension in sitting using weighted ankle bands.

Participants in the treatment group will receive a minimum of three follow-up telephone calls from the physiotherapist during the twelve week intervention period. The physiotherapist will use the calls to encourage and monitor adherence, discuss any adaptations or progressions to exercises and to review goals [47]. Participants will be asked to complete a daily exercise diary to monitor their adherence to the programme and to record any adverse events or barriers to exercise they encounter.

#### Adverse event reporting

Participants in both groups will be asked to contact either the principal investigator or research physiotherapist if they experience any adverse events during the study/intervention period. All events will be recorded and reviewed by the principal investigator and the participant’s GP informed if appropriate. Serious adverse events will be referred to the study sponsor and a monitoring group will be convened in order to review the event and ongoing trial safety. Adverse events will be included as a secondary outcome of the study.

### Outcome assessment

#### Primary outcome

*Activity limitation* will be assessed using the recently validated Rasch based Overall Disability Scale (RODS) [48]. This 24-item interval scale for activity limitation has greater responsiveness than previous ordinal measures [49]. Scores for the RODS will be collected at baseline, at the end of the twelve week intervention period and at a twelve month follow up.

#### Secondary outcome measures

All of the following secondary outcome measures will be assessed at baseline, twelve weeks and twelve months:

*Activity limitation* will also be assessed as a secondary outcome using the Overall Neuropathy Limitations Scale (ONLS) [34]. This is a 12 item ordinal scale of activity limitation developed from the ODSS, which was used to calculate sample size for the present study [35].

*Fatigue* will be assessed using the Rasch-modified Fatigue Severity Scale (RFSS) [50]. This seven-item scale is a Rasch-built modified version of the Fatigue Severity Scale, and was developed specifically to address fatigue in individuals with immune-mediated neuropathy. The authors report good psychometric properties for the scale.

*Psychological wellbeing* will be assessed using the Hospital Anxiety and Depression Scale (HADS) [51]. The scale consists of separate anxiety and depression subscales of seven items each and has been found to correlate well with other measures of depression and anxiety.

*Functional health and well being* will be assessed using the Medical Outcomes Short Form 12 (SF-12). The norms-based scoring system allows direct data comparisons to be made between the SF-12 and other generic health surveys [52, 53].

*Levels of physical activity* will be assessed using the 7-question International Physical Activity Questionnaire (IPAQ-short). The questions ask participants to report the frequency and duration of moderate activity, vigorous activity and walking, and to rate the proportion of time they spent sedentary in the previous week. The scale has shown acceptable measurement properties, similar to other self-report assessments [54].

*Health beliefs* will be assessed using the Brief Illness Perceptions Questionnaire (Brief-IPQ) [55] developed from the revised Illness Perceptions Questionnaire (IPQ-R) [56]. Nine questions address participants beliefs with regard to various features such as perceived severity of consequences, perceived chronicity of an illness or condition, and perceived control over the course or treatment of a given condition. Participants respond on an eleven point Likert scale (for example, 0 = no symptoms at all, 10 = many severe symptoms).

*Self-efficacy;* an individuals confidence in their ability to exercise, will be measured using the self-efficacy for exercise (SEE) scale [57]. The scale examines 9 potential barriers to exercise, such as feeling tired or depressed; participants are asked to rate each item on an eleven point Likert scale spanning the extent to which they feel confident that they could exercise in the presence of a particular barrier (0 = Not confident, 10 = Very confident). The authors report good internal consistency (alpha 0.92) and good predictive validity for the scale [57].

*Adherence* to the tailored home exercise programme in the study will be evaluated using a) the Exercise Adherence Rating Scale (EARS) and b) self-report diary entries.

- The EARS has been developed based on the Medication Adherence Rating Scale [58] and evaluates barriers to adherence for prescribed exercise by asking participants to rate, on a five-point Likert scale, the extent to which they agree with sixteen statements about their exercise adherence (for example, “I forget to do my exercises”, “I’m not sure how to do my exercises”). The EARS will only be completed by participants randomised to the tHEP intervention.
- Self-report exercise diaries record exercise type, completion and factors affecting exercise. The number of repetitions of strengthening exercise and duration of aerobic exercise will be used to calculate overall adherence as a proportion of the exercise prescribed.

### Cost-effectiveness and cost-utility analyses

In order to evaluate the cost-effectiveness and cost-utility of the intervention in relation to advice and usual care, participants in both groups will complete the Client Services Receipt Inventory (CSRI) and the EQ-5D at baseline and twelve month follow-up.

The CSRI is a scale developed for collecting data relating to service use and has been used in a large number of evaluations of health and social care interventions over the past 30 years [38]. The questionnaire records the interviewee’s use of health and social care services, accommodation and living situation, income, employment and benefits. Service use data are combined with appropriate unit costs [59] to generate total care costs per patient. Lost employment will be valued using average wage rates.

The EQ-5D [60] is a brief scale designed to measure health related quality of life across five dimensions: mobility, self-care, usual activities, pain/discomfort and anxiety/depression. For each domain participants are asked to rate their current health using a five point scale to describe the extent to which they can perform activities (“No problems” to “Unable to perform”). The scale also includes a visual analogue scale on which participants mark their current health status between 0 (worst health they can imagine) and 100 (best health they can imagine). Quality Adjusted Life Years (QALYs) will be derived from the EQ-5D scores for the cost-utility analysis.

### Qualitative assessment of intervention acceptability

A purposive sub-sample of 8–10 participants from the tHEP group and up to 5 from the usual care group will be invited to attend a semi-structured interview following completion of their twelve month follow-up to evaluate participants’ experiences. Participants in the tHEP group will be asked about their experience of the tHEP and the factors that facilitate and impede adherence to it. Participants in the usual care group will be asked whether the advice they received prompted any change in exercise or physical activity behaviour and about their experience of exercise. The interviews will be recorded, transcribed and undergo thematic analysis to explore themes within the data [61].

### Data analysis

#### Efficacy of intervention

For our primary outcome of activity limitation (RODS) we will adjust for baseline score. For all other outcomes, we will adjust for the severity stratification group using ONLS baseline score, but not for the IVIg stratification group as the likely low numbers will lead to an unstable model.

Intention to treat analysis will be our primary model for efficacy analysis. We will conduct a linear regression of change from baseline to the twelve month follow up and add in both intervention arm and baseline score. A secondary analysis will examine changes between baseline and 12 week follow up. Complete case analysis is most often used but not ideal in this study as we have small numbers. If we have some missing data at twelve month follow up we can attempt to extrapolate based on the data from participants with complete datasets if there is less than 25 % missing data.

Per protocol analysis: If we can identify a suitable cut-off from our measure of adherence we can also run a per protocol analysis by removing those participants from the dataset who did not complete enough of the exercise intervention to be able to show an effect. However, due to low numbers we do not wish to put the analysis at risk by excluding too many.

We will also run an exploratory secondary subgroup analysis of the self-efficacy data in order to see whether self-efficacy influences the efficacy of the intervention. A high/low score threshold will be identified for this purpose.

#### Cost-effectiveness and cost-utility analyses

Costs generated from the CSRI will be added to costs of the intervention. These will be derived from information on staff time involved in delivering the intervention and other non-staff costs. The endpoint for the economic analysis will be twelve months when, if costs are lower in the exercise group and outcome better, then the exercise intervention will have been cost-effective. However, if costs are higher and outcomes better then we will construct cost-effectiveness acceptability curves to show the probability that the intervention is cost-effective for different values placed on a change in outcome. Cost-utility analyses will be conducted in a similar way, but will use QALYs as the outcome measure. Uncertainty around the results will be explored using cost-effectiveness planes and acceptability curves.

#### Participant experience and factors influencing adherence

Data from transcriptions of semi-structured interviews will be analysed thematically to identify common themes and connections between themes.

## Discussion

This is the first randomised controlled trial to compare the efficacy and cost-effectiveness of a tHEP with advice about exercise and usual care in IN. A feasibility study by this research group [24] provided evidence that tailored home exercise is acceptable to individuals with IN and that participation in a tHEP was associated with significant improvements in activity limitation, fatigue, quality of life and mood in an uncontrolled study.

There are two key strengths of the current RCT: firstly, the integrity and quality of the intervention delivery is enhanced by having one physiotherapist to conduct all assessments and exercise prescription that includes the use of behavioural change techniques to facilitate uptake and adherence to the exercise, and second the qualitative analysis of interviews with participants after completion of the intervention, and the cost-effectiveness and cost-utility analyses, will provide valuable information regarding the acceptability and feasibility of the intervention as a potential standard of care for individuals experiencing activity limitation and participation restriction as a result of stable IN.

## Abbreviations

IN: Inflammatory neuropathy
UC: Usual care
tHEP: Tailored home exercise programme
GBS: Guillain-Barré syndrome
CIDP: Chronic inflammatory demyelinating polyradiculoneuropathy
PDN: Paraproteinaemic demyelinating neuropathy
TTM: Transtheoretical model
